# Oral hygiene practices and associated factors among rural communities in northwest Ethiopia

**DOI:** 10.1186/s12903-024-04049-4

**Published:** 2024-03-09

**Authors:** Zemichael Gizaw, Negesu Gizaw Demissie, Mulat Gebrehiwot, Bikes Destaw Bitew, Adane Nigusie

**Affiliations:** 1 Department of Environmental and Occupational Health and Safety, Institute of Public Health, College of Medicine and Health Sciences, University of Gondar, Gondar, Ethiopia; 2 Department of Medical Nursing, School of Nursing, College of Medicine and Health Sciences, University of Gondar, Gondar, Ethiopia; 3 Department of Health Education and Behavioral Sciences, Institute of Public Health, College of Medicine and Health Sciences, University of Gondar, Gondar, Ethiopia

**Keywords:** Oral health, Oral hygiene, Mouthwash, Teeth cleaning, Toothbrush sticks, Gum pricking, Rural communities, Ethiopia

## Abstract

**Background:**

Poor oral hygiene affects the overall health and quality of life. However, the oral hygiene practice in rural communities and contributing factors are not well documented. Accordingly, this study was conducted to assess oral hygiene practices and associated factors among rural communities in northwest Ethiopia.

**Methods:**

A cross-sectional study was conducted among 1190 households. Data were collected using a structured and pretested questionnaire, prepared based on a review of relevant literature. The questionnaire comprises socio-demographic information, access to health and hygiene messages, oral hygiene practices, and water quality. We assessed oral hygiene practices with these criteria: mouth wash with clean water in every morning, mouth wash with clean water after eating, brushing teeth regularly, and avoiding gum pricking. Gum pricking in this study is defined as sticking needles or wires into gums to make the gums black for beauty. Multivariable logistic regression was used to identify factors associated with oral hygiene practices. Significant associations were declared on the basis of adjusted odds ratio with 95% confidence interval and *p*-values < 0.05.

**Results:**

Results showed that all the family members usually washed their mouth with clean water in everyday morning and after eating in 65.2% and 49.6% of the households, respectively. Furthermore, 29.9% of the households reported that all the family members regularly brushed their teeth using toothbrush sticks and one or more of the family members in 14.5% of the households had gum pricking. Overall, 42.9% (95% CI: 39.9, 45.6%) of the households had good oral hygiene practices. Health and/or hygiene education was associated with good oral hygiene practices in the area (AOR: 1.66, 95% CI: 1.26, 2.21).

**Conclusion:**

More than half of the households had poor oral hygiene practices in the area and cleaning of teeth with toothpastes is not practiced in the area, where as gum pricking is practiced in more than one-tenth of the households. The local health department needs provide community-level oral health education/interventions, such as washing mouth with clean water at least twice a day, teeth brushing using indigenous methods such as toothbrush sticks or modern methods such as toothpastes, and avoiding gum pricking to promote oral health.

**Supplementary Information:**

The online version contains supplementary material available at 10.1186/s12903-024-04049-4.

## Background

Oral health is a critical component of overall body health and an important factor in an individual’s overall well-being. A healthy mouth with a disease-free oral cavity and its surrounding structures constitutes good oral health. Like other areas of the body, mouth teems with bacteria, mostly harmless. But mouth is the entry point to digestive and respiratory tracts, and some of these bacteria can cause disease. Normally the body’s natural defenses and good oral hygiene, such as daily brushing and flossing, keep bacteria under control [1–3].

However, without proper oral hygiene, bacteria can reach levels that might lead to oral infections, such as tooth decay and gum disease [4, 5]. Oral diseases are estimated to affect nearly 3.5 billion people at global level [6] and the 2019 global disease burden estimate showed that about 2 billion people worldwide suffer from permanent tooth caries, with 520 million children suffering from primary tooth caries and approximately 14% of the global adult population, representing to more than one billion cases worldwide are affected by periodontal diseases [6]. Moreover, oral diseases have also significant economic consequences, which include direct, indirect, and intangible costs such as treatment costs, missed school and work days, and decreased quality of life [7]. For instance, dental diseases (excluding oral and pharyngeal cancers) costed approximately $545 billion US dollar in 2015 [8].

Maintaining oral hygiene at good condition is an important day-to-day activity to prevent poor oral hygiene associated health problems. Indigenous and modern methods are available to maintain oral health. The use of traditional means of oral hygiene such as plant-based traditional toothbrush sticks has been used to maintain oral hygiene good and to treat oral diseases as documented in literature [9–12]. The use of toothbrush sticks (in many cases also known as chewing sticks) is widespread in Ethiopia, both for esthetic and hygienic purposes. In Ethiopia, a chewing stick, generally called the “mefakia’’. The use of toothbrush sticks to maintain oral hygiene is also recommended by world health organization [13]. A toothbrush stick is generally obtained from any slim woody part of trees. Mostly it is harvested from branches although harvest from woody roots is also known. Some of the common plants used for toothbrush sticks in Ethiopia are Akeya (*Salix subserrata*), Weira (*Olea africana*), Kacha (*Agave sisolana*), Kechemo (*Myrsine africana*), Zembaba (*Phoenix reclinata*), Chifrig (*Sida cunefolia*), and Limitch (*Clausena anisata*) [13]. Toothbrush sticks contain an antiseptic property and have no plaque deposits and toxicity [14–16]. Moreover, tooth brushing using toothpastes, flossing, and other healthy lifestyle measures such as minimizing tobacco use and sugary intake are the most recommended measures to maintain oral health. Teeth brushing twice a day using toothpastes (one in the morning and second before going to sleep at the night) is the primary way to maintain good oral hygiene. Fluoride, a common ingredient in toothpaste helps prevent cavities. Moreover, the antiseptic nature of toothpastes can limit growth of microbes and the mechanical action of brushing helps to remove solid particles [17–19]. However, brushing does not remove all the solid particles from teeth. Therefore, flossing with thorough rinsing by clean water plays an important role in removing all the small particles from the teeth [20–22]. Health lifestyles such as avoiding or minimizing tobacco use, soda drinks, and sugary intakes play a remarkable contribution to keep the oral cavity healthy. Tobacco intake increases the plaque level in the teeth and weakens the teeth [23–25]. Soda drinks cause teeth damage [26–28].

Despite indigenous and modern methods are available to maintain oral health, significant oral health disparities exist in rural communities, especially in developing countries. These disparities result from a number of factors including low priority to oral health, geographic isolation, cultural norms, poverty, oral health illiteracy, and other contextual factors such as deficient infrastructures, underprovided public services and unequal distribution of health services [29–32]. However, oral hygiene practices and contextual factors in the rural northwest Ethiopia is not documented and there is still minimal research on the oral health of rural populations in the area. This study was, therefore, conducted to assess oral hygiene practices and associated factors among rural communities in northwest Ethiopia.

## Methods

### Study design and setting

A community-based cross-sectional study was conducted among rural households in central and north Gondar administrative zones of the Amhara national regional state, Ethiopia in May 2016. North Gondar zone covers seven woredas and is bordered on the south by central Gondar zone, on the north by the Tigray region, and on the east by Wag Hemra zone. Debarq town is the capital city of the zone [33]. The total population in north Gondar zone is estimated to be 912,112 [34]. Central Gondar zone covers thirteen woredas and is bordered on the south by Lake Tana, west Gojjam zone, Agew Awi zone and the Benishangul-Gumuz region, on the west by west Gondar zone, on the north by the Tigray region and north Gondar zone, on the east by Wag Hemra zone and on the southeast by south Gondar zone [35]. Gondar city is the capital city of central Gondar zone. The total population in central Gondar zone is estimated to be 2,896,928 [34].

### Sample size calculation and sampling procedures

The sample size was calculated using simple population proportion formula with the following assumptions: proportion of rural households who had good oral hygiene (p) = 50% since there was no similar study in the area, level of significance (α) = 5%, 95% confidence interval (standard normal probability), z: the standard normal tabulated value, and margin of error (d) = 5%.

$$n= \frac{{Z\alpha }^{2}p(1-p)}{{d}^{2}} = \frac{{1.96}^{2}*0.5(1-0.5)}{{0.05}^{2}} = 384$$

The final sample size was 1210, with a design effect of 3 and a non-response rate of 5%. All rural households in central and north Gondar administrative zones were considered for sampling. First, we chose 4 districts or woredas out of 22 using lottery method and we then selected 7 kebeles (the lowest administrative unit in Ethiopia) from each district at random using a simple random sampling technique, that is, the lottery method. Finally, we selected 1210 rural households (the analysis unit of this study) using a systematic random sampling technique. Forty-three households were included in each kebele (the number of households in each kebele was determined by equally devising the total sample size to each kebele). We began collecting data in households located on the right side of the local administrators’ office. Assuming that the average number of households in each rural kebele is 200 [36, 37], a sampling interval (K = 5) was calculated by dividing 200 by the kebele’s predetermined sample size (*n* = 43). Following that, a number between one and the sampling interval was chosen at random using the lottery method, which is known as the random start, and was used as the first number included in the sample. Then, after the first random start, every fifth household was sampled until the desired sample size for each kebele was reached.

### Data collection tools and procedures

A structured and pretested questionnaire was used to collect data, prepared based on a review of relevant literature [38, 39]. The questionnaire was first prepared in English language and translated to the local Amharic language, and back translated into English to check consistency. The questionnaire comprises socio-demographic information, access to health and sanitation messages, oral hygiene practices, and water quality (Supplementary file 1). Environmental health experts were participated in the data collection process. We provided training for the data collectors, provided them with a guide for the questionnaire, and field supervisors closely supervised the data collection process and checked completeness of data in each day of data collection to improve inter and intra interviewers’ reliability during the interview. The training was about each item in the questionnaire, interview techniques, and ethical issues during interview.

### Measurement of outcome variable

Oral hygiene practices of households, the primary outcome variable of the study, was taken as “good” if all the family members wash their mouth with clean water in everyday morning after getting from bed, wash/rinse their mouth with clean water after eating, regularly brush or clean their teeth with toothbrush sticks, and if the family members have no traditional gum pricking. Gum pricking in the current study is sticking needles or wires into gums to make the gums black for beauty.

### Data processing and analysis

Data were entered into EPI-INFO version 3.5.3 and exported to Statistical Package for Social Sciences (SPSS) version 20 for further analysis. For most variables, data were presented by frequency and percentage. We included variables to the multivariable binary logistic regression model from the literature regardless of their bivariate *p*-value to identify factors associated with oral hygiene practices of rural households. Statistically significant association was declared on the basis of adjusted odds ratio (AOR) with 95% confidence interval (CI) and *p*-values < 0.05. Model fitness was check using Hosmer and Lemeshow goodness-of-fit test.

## Results

### Characteristics of study households

Of a total of 1210 rural households, 1190 households participated in the current study, with a response rate of 98.3%. The mean ($\pm$SD) family size was 5 ($\pm$2) and 513 (43.1%) of the households had family size more than the mean. Two hundred and ninety-two (24.7%) and 442 (40.7%) of the female and male household heads, respectively attended formal education. Rural households accessed hygiene and sanitation messages via health education [565 (47.5%)], health supervision [967 (81.3%)], and family discussion [812 (68.2%)]. Almost all, 1154 (97%) of the households had no basic access to drinking water, i.e., 20 l/c/d (Table 1).

**Table 1:** Characteristics of study households (*n* = 1190) in a rural setting of northwest Ethiopia, May 2016

Variables	Frequency	Percent
Family size		
$\le$ 5	677	56.9
$>$ 5	513	43.1
Maternal education (*n* = 1180)		
No formal education	888	75.3
Attend formal education*	292	24.7
Paternal education **(***n* = 1085)		
No formal education	643	59.3
Attend formal education*	442	40.7
The household receive health and hygiene education in the last three months		
Yes	565	47.5
No	625	52.5
Health extension workers regularly supervise health and hygiene conditions of the household		
Yes	967	81.3
No	223	18.7
The family regularly discusses about health issues including oral hygiene		
Yes	812	68.2
No	378	31.8
Volume of water collected per day		
$<$ 20 l/c/d	1154	97.0
$\ge$ 20 l/c/d	36	3.0

l/c/d: Liter per capita per day

*formal education includes primary and secondary education.

### Oral hygiene practices

**Fig. 1:**
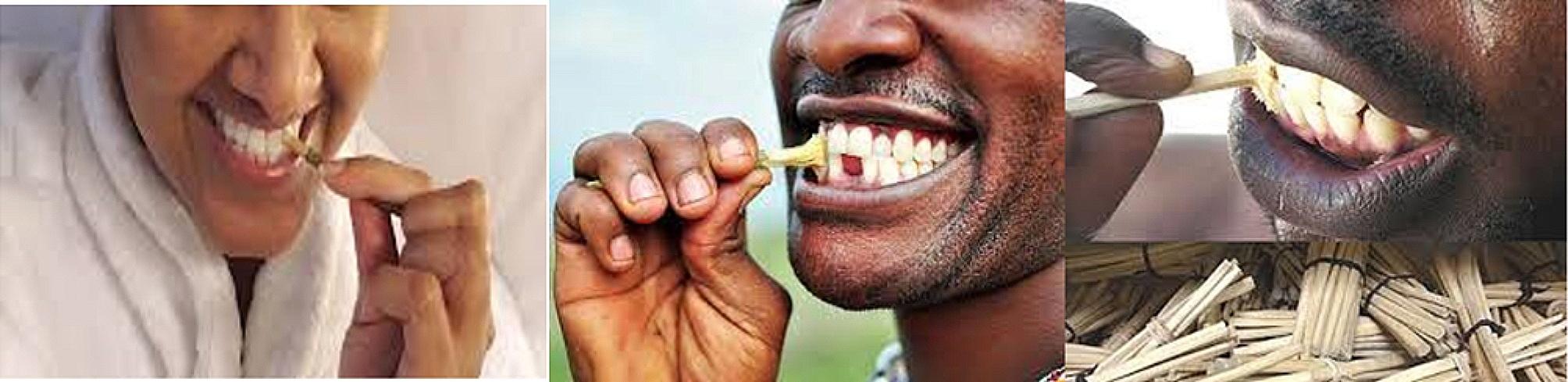
Photos showing the use of toothbrush sticks to brush teeth. (source: free google images)

About two-third, 776 (65.2%) of the households reported that all the family members usually washed their mouth with clean water in everyday morning and 590 (49.6%) of the households reported that all the family members usually washed their mouth with clean water after eating. Furthermore, 356 (29.9%) of the households reported that all the family members regularly scrub their teeth using toothbrush sticks. Figure 1 illustrates the use of toothbrush sticks in the studied region. One hundred and seventy-three (14.5%) of the households reported that one or more family members had gum pricking. Overall, 510 (42.9%) (95% CI: 39.9, 45.6%) of the households had good oral hygiene practices (Table 2).

**Table 2:** Oral hygiene practices among households (*n* = 1190) in a rural setting of northwest Ethiopia, May 2016

Variables	Frequency	Percent
All the family members wash their mouth with clean water in everyday morning		
Yes	776	65.2
No	414	34.8
All the family members wash their mouth with clean water after eating		
Yes	590	49.6
No	600	50.4
Do all the family members scrub their teeth using toothbrush sticks		
Yes	356	29.9
No	834	70.1
Traditional gum pricking		
Yes	173	14.5
No	1017	85.5
Oral hygiene		
Poor hygiene	680	57.1
Good hygiene	510	42.9

### Factors associated with oral hygiene

Health and/or hygiene education, health supervision by community health workers, family discussion about hygiene and sanitation, volume of water collected per day, maternal education, paternal education, and family size were all the variables entered into the multivariable binary logistic regression model regardless of their *p*-values in the bivariate analysis. In the adjusted model, only health and/or hygiene education was statistically associated with oral hygiene practices of rural households. Households who received health and/or hygiene education in the last three months prior to the survey had 1.66 times more odds to have good oral hygiene practices compared with households who didn’t receive health and/or hygiene education (AOR: 1.66, 95% CI: 1.26, 2.21) (Table 3).

**Table 3:** Factors associated with oral hygiene practices among households (*n* = 1190) in a rural setting of northwest Ethiopia, May 2016

Variables	Oral hygiene		COR with 95% CI	AOR with 95% CI
	Good	Poor		
The household receive health and hygiene education in the last three months				
Yes	272	293	1.51 (1.20, 1.90)	1.66 (1.26, 2.21)***
No	238	387	1.0	1.0
Health extension workers regularly supervise health and hygiene conditions of the household				
Yes	411	556	0.93 (0.69, 1.24)	0.76 (0.53, 1.09)
No	99	124	1.0	1.0
The family regularly discusses about health issues including oral hygiene				
Yes	360	452	1.21 (0.94, 1.55)	1.09 (0.80, 1.49)
No	150	228	1.0	1.0
Maternal education				
No formal education	371	517	1.0	1.0
Attend formal education	134	158	1.18 (0.91, 1.54)	1.27 (0.94, 1.72)
Paternal education				
No formal education	272	371	1.0	1.0
Attend formal education	189	253	1.02 (0.80, 1.30)	0.91 (0.69, 1.19)
Family size				
$\le$ 5	286	391	0.94 (0.75, 1.19)	0.89 (0.69, 1.14)
$>$ 5	224	289	1.0	1.0
Volume of water collected per day				
$<$ 20 l/c/d	497	657	1.0	1.0
$\ge$ 20 l/c/d	13	23	0.75 (0.38, 1.49)	0.85 (0.38, 1.91)

Note: *** statistically significant at *p* < 0.001, Hosmer and Lemeshow test = 0.982, AOR: Adjusted odds ratio, CI: Confidence interval, COR: Crude odds ratio

## Discussion

This is a community-based cross-sectional study conducted to assess oral hygiene practices of rural households in northwest Ethiopia and found that 42.9% (95% CI: 39.9, 45.6%) of the households had good oral hygiene practices. This finding is comparable with findings of studies among rural populations in India, 42% [1]. On the other hand, the good-level practice of oral hygiene in the current study is lower than the good-level practice of oral hygiene reported by studies among rural dwellers in Delta and Edo State of Nigeria, 66.2% [40], a rural areas of Kachchh district of India, 81% [41], rural villages of 23 states of India 83% [42], and Dehradun district of India 50% [43]. The lower level of oral hygiene practices in the studied region can be explained by lower oral health literacy. Poor health literacy can result in poor oral hygiene and difficulty in using different oral health measures. Rural residents with low health literacy are more likely to practice bad habits that affect oral health such as pricking and tobacco use. Moreover, extreme poverty in the area may explain poor oral hygiene. In poverty, survival may naturally take precedence over oral hygiene. Hygiene promotion may not be immediate enough for attention beyond pressing needs, for example, the need for food and the means to produce it. In addition, oral health is considered as a much lesser priority in Ethiopia, especially in the rural areas. Due to limited resources available to the health sector, assignments are mainly directed towards life threatening health conditions rather than oral hygiene.

Oral health is fundamental to overall health. The health of our mouth, teeth, and gums can affect our general health [44, 45]. Our oral health might contribute to various diseases and conditions, including endocarditis (this infection of the inner lining of your heart chambers or valves typically occurs when bacteria or other germs from another part of our body, such as from mouth, spread through our bloodstream and attach to certain areas in our heart) [46, 47], cardiovascular disease (heart disease, clogged arteries, and stroke might be linked to the inflammation and infections that oral bacteria can cause) [48, 49], diabetes and pancreatic cancer (gum disease causes inflammation, which makes it harder for your body to use insulin properly. Gum disease can also contribute to certain types of cancer, especially pancreatic cancer) [50–52], pregnancy and birth complications (periodontitis has been linked to premature birth and low birth weight) [53, 54], and pneumonia (certain bacteria in our mouth can be pulled into our lungs, causing pneumonia and other respiratory diseases) [55, 56]. Therefore, practicing good oral hygiene offers advantages that go beyond cavity prevention. Some of the benefits of good oral hygiene include healthier gums, reduced risk for heart attack, healthier lungs, lower chances of diabetes, decreased cancer risk, and safer pregnancy.

While it is common in industrialized countries to use factory made toothbrushes, most of the rural populations in Ethiopia use toothbrush sticks to maintain oral hygiene. Toothbrush sticks can be used by the vast majority of people in Ethiopia who cannot afford to buy the commercial toothbrush and toothpaste. The cleansing efficacy of traditional toothbrush sticks is achieved by the mechanical effects of the stick fibers, antimicrobial constituents of the trees, and a combination of mechanical and chemical actions [57]. However, some toothbrush sticks may have some negative side effects such as teeth discoloration if used for an extended period of time. The rough fibers may also have undesirable effect of scratching the teeth enamel and worse bleeding the gums to allowing bacteria in [14].

This study also explored that health and/or hygiene education was significantly associated with oral hygiene practices in the studied region. Households who received health and/or hygiene education in the last three months prior to the survey had more odds to have good oral hygiene practices. This could be due to the fact that health and/or hygiene education encourages changes in healthy behaviors. Moreover, health and/or hygiene education is an effective strategy to create demand for self-care and thereby increase practices of good oral health measures. Health and/or hygiene education disseminates health information and vital skills necessary to adopt practices and maintain health-enhancing behaviors. Health and/or hygiene education also enables people to take actions to improve their health [5, 58–60].

To our knowledge, no studies have assessed oral hygiene practices and associated factors among rural communities in Ethiopia. The study used structured and pretested data collection and the data collection was closely supervised to increase quality of data and completeness of the questionnaire. Moreover, study subjects were selected at random using systematic random sampling technique and so that all the rural households in the study area had an equal chance to be included in the study and findings of this study will be generalizable. The results of this study could be, therefore, useful in the development of programs for oral health promotion for rural residents and in the development of collaborative rural research activities in the field of oral health. However, the self-reported data may not be reliable to measure oral hygiene since the study subjects may make the more socially acceptable answer rather than being truthful and they may not be able to assess themselves accurately. Moreover, we did not adjust for psychological or behavioral factors which are linked to oral hygiene practice [61, 62].

## Conclusion

In the study area, 42.9% of the households had good oral hygiene practices and more than half of the households had poor oral hygiene practices. Cleaning of teeth with toothpastes is not practiced in the area and one or more of the family members in more than one-tenth of the households practiced gum pricking. Health and/or hygiene education was found to be significantly associated with oral hygiene in the studied region. The local health department needs provide community-level oral health education to promote oral hygiene in the community and encouraging the community to use different interventions such as washing mouth with clean water at least twice a day, teeth brushing using indigenous methods such as toothbrush sticks or modern methods such as toothpastes and avoiding gum pricking to promote oral health.

### Electronic supplementary material

Below is the link to the electronic supplementary material.

Supplementary Material 1

## Data Availability

Data will be made available upon requesting ZG, the primary author of this study.

## Abbreviations

AOR: Adjusted odds ratio
CI: Confidence interval
COR: Crude odds ratio
l/c/d: Liter per capita per day
SD: Standard deviation
SPSS: Statistical package for social sciences
