# Supplementary material for: Oral hygiene practices and associated factors among rural communities in northwest Ethiopia
Source: BMC Oral Health. 2024 Mar 9;24:315. doi: 10.1186/s12903-024-04049-4 (PMC10924987; doi:10.1186/s12903-024-04049-4)
Supplement: Supplementary file 1 — Supplementary Material 1 [file 12903_2024_4049_MOESM1_ESM.doc]

| **Questionnaire Identification Code:** | **District:** | **Kebele:** | **HH:** |
| --- | --- | --- | --- |

**Consent Form**

Hello, I am ________________________ working with a research team from University of Gondar. We are contacting you to collect information for a study conducted to Oral hygiene practices and associated factors among rural communities in northwest Ethiopia. This questionnaire is prepared to collect information from households about Oral hygiene practices and associated factors.

Thank you so much for agreeing to be interviewed for this project. All of the information we got from you will be completely confidential and coded with unique number. Your name will not be written down and will never be used in connection with any of the information you provide. If you do not want to answer all or some of the questions, you have the right to refuse participation at any time. However, we would greatly appreciate your help in responding to this questionnaire. This questionnaire is expected to be completed in 20 - 30 minutes.

Do I have your permission to continue?

1. **Yes,** continue your interview with thanks
2. **If no,** skip to the next participant by writing reasons for his/ her refusal;

____________________________________________________________________________

**Data Collector:** Name: ____________________Signature:___________Date: __________

Time started _______Time completed_______

**Result of interview**:

1. Completed 3. Refused

2. Respondent not available 4. Incomplete

**Approval from Supervisor:**

Name: ___________________ Signature: ___________ Date: ________________

| **Part 1: Demographic information** | | | | | | | | | | | | | | | | |
| --- | --- | --- | --- | --- | --- | --- | --- | --- | --- | --- | --- | --- | --- | --- | --- | --- |
|  | List of Family member | Sex | Age | | | Education | Marital status | Occupation | | | | | Family Head | | | |
|  |  |  |  |  |  |  |  |  |  |  |  |  |  |  |  |  |
|  |  |  |  |  |  |  |  |  |  |  |  |  |  |  |  |  |
|  |  |  |  |  |  |  |  |  |  |  |  |  |  |  |  |  |
|  |  |  |  |  |  |  |  |  |  |  |  |  |  |  |  |  |
|  |  |  |  |  |  |  |  |  |  |  |  |  |  |  |  |  |
|  |  |  |  |  |  |  |  |  |  |  |  |  |  |  |  |  |
|  |  |  |  |  |  |  |  |  |  |  |  |  |  |  |  |  |
| **List of Family member**   1. Father 2. Mother 3. Son (if more than one wrte S1, S2, S3, …) 4. Daughter (if more than one write D1, D2, D3 …) 5. Relative (if more than one, write R1, R2, …) 6. House maid 7. Grandfather 8. Grand mother | | Sex   1. Male 2. Female | | Head of the family   1. Father 2. Mother 3. Grand father 4. Grand mother 5. Old son 6. Old daughter | | **Marital status**   1. - Married 2. Single 3. Divorced 4. Separated 5. Widowed 6. Cohabited 7. Not applicable | | **Educational status**   1. Pre-school 2. Not read and write 3. Read and write 4. Primary school 5. Secondary school 6. 12 complete 7. College/University | | | | | **Occupation**   1. Farmer 2. Merchant 3. Civil servant 4. Student 5. NA | | | |
| **Part 2: Health information and supervision** | | | | | | | | | | | | | | | | |
|  | Have you discused about health, oral hygiene, sanitation and other health issues with the family members? | | | | 1. Yes 2. No | | | | | | | | | |  | |
|  | Have you discused about health, oral hygiene, sanitation and other health issues with your 1:5 comminity organization members? | | | | 1. No organization 2. Yes 3. No | | | | | | | | | |  | |
|  | Are you frequently supervised by the community/ kebele leaders? | | | | 1. Yes 2. No | | | | | | | | | |  | |
|  | Does the health extension worker or other health professional closely supervise you? | | | | 1. Yes 2. No | | | | | | | | | |  | |
|  | Have you heard any health/hygiene messages for the last 3 months? | | | | 1. Yes 2. No | | | | | | | | | |  | |
|  | If ‘Yes’ for # 5, from where you heard? [Multiple answer is posible] | | | | 1. Government’s health workers 2. Community Health Volunteers 3. School children 4. NGO staff 5. Church/Mosque 6. Poster/flyer/leaflets 7. Radio 8. TV 9. Community events 10. Private/ community discussion 11. Family discussion 12. Clinic/hospital 13. Traditional leader 14. Others __________________________ | | | | | | | | | |  | |
|  | If ‘Yes’ for # 5, can you tell me which health/hygiene/ sanitation messages you can recall? | | | | __________________________________  ____________________________________  _______________________________________ | | | | | | | | | |  | |
| **Part 3: Oral hygiene** | | | | | | | | | | | | | | | | |
|  | When all the family members wash their mouth with clean water | | | | 1. No [ write number ] 2. Every morning after getting from bed [ write number ] 3. Every 2 -3 days after getting from bed [ write number ] 4. Every 4-5 days after getting from bed [ write number ] 5. Every 6-7 days after getting from bed [ write number ] 6. After food [ write number ] | | | | | |  | | | | | |
|  | How frequently all the family members regularly brush or clean their teeth with chop sticks or tooth cleaner? | | | | 1. Never [ write number ] 2. Every day [ write number ] 3. Every 2 -3 days [ write number ] | | | | | |  | | | | | |
|  | Do the family members share chop sticks/ tooth brushes used by another person? | | | | 1. Yes 2. No | | | | | |  | | | | | |
|  | How many of the family members had traditional gum pricking for the purpose of beauty? | | | | __________________ | | | | | |  | | | | | |
|  | Kindly give me the key times you and other family members usually wash your hands? (more than one answer is posible) | | | | Pick time | | | | Yes | | | No | | | |  |
|  |  |  |  |  | Before eating | | | |  | | |  | | | |  |
|  |  |  |  |  | After latrine use | | | |  | | |  | | | |  |
|  |  |  |  |  | After handling baby’s diaper/feces | | | |  | | |  | | | |  |
|  |  |  |  |  | After eating | | | |  | | |  | | | |  |
|  |  |  |  |  | Before feeding child | | | |  | | |  | | | |  |
|  |  |  |  |  | Before food preparation | | | |  | | |  | | | |  |
|  |  |  |  |  | After handling rubbish/ animals | | | |  | | |  | | | |  |
|  |  |  |  |  | Others, specify ______________ | | | | | | | | | | | |
|  | What do you usually use in washing hands? (more than one answer is posible) | | | | 1. Water only 2. With Soap 3. With Sand/leaves 4. With ash 5. Other, specify ________ | | | | |  | | | | | | |
|  | If the answer is water only, what is the main factor that prevents your family from using soap? | | | | 1. Negligence 2. Washing with soap takes time 3. Water alone cleanses the hand 4. Expensive to buy soap 5. Other, specify _______________ | | | | |  | | | | | | |
|  | Do you and your family members cut finger nails with their teeth? | | | | 1. Yes [write number of the family who do this ] 2. No [write number ] | | | | |  | | | | | | |
|  | Do all the family members wash their face with clean water and/or soap in the morning immediately after getting up from bed? | | | | 1. Yes [write number ] 2. No [write number ] | | | | |  | | | | | | |
|  | Do all the family members wash their face with clean water and/or soap after return from work? | | | | 1. Yes [write number ] 2. No [write number ] | | | | |  | | | | | | |
| **Part 4: Water quality and safety measures** | | | | | | | | | | | | | | | | |
|  | What is the main source of water for your household? | | | | 1. Piped line 2. Public taps/tapstand/standpipes 3. Protected well 4. Unprotected well 5. Protected spring 6. Unprotected spring 7. Protected Rain catchment 8. Unprotected Rain Catchment 9. Surface water (river, dam, lake, ponds, etc.) 10. Others, specify ____________________ | | | | | | | | |  | | |
|  | How many liters of water do the entire households consume/use per day? (Note: Jerry Can = 5L, 10L, 20L and pot = 30 L ) | | | | _________________________ | | | | | | | | |  | | |
|  | Observe the condition of the water containers for collection and storage. | | | | 1. Covered 2. Not covered 3. Others are covered while some are not | | | | | | | | |  | | |
|  | Do you treat your water? | | | | 1. Yes 2. No | | | | | | | | |  | | |
|  | If ‘Yes’ for #20, which treatment do you use regularly? (more than one answer is posible) | | | | 1. Solar disinfection 2. chlorine/waterguard/aquatab/bleach/ wuha agar/ bishan gari 3. Boiling 4. Cloth/ sieve filtration 5. Sedimentation 6. Others, specify ______________ | | | | | | | | |  | | |

**I have finished my interview, thank you for your cooperation!!!**
